# Neutrophil trafficking to lymphoid tissues: physiological and pathological implications

**DOI:** 10.1002/path.5227

**Published:** 2019-02-04

**Authors:** Mathieu‐Benoit Voisin, Sussan Nourshargh

**Affiliations:** ^1^ Centre for Microvascular Research, William Harvey Research Institute, Barts and the London School of Medicine and Dentistry, Queen Mary University of London London UK

**Keywords:** neutrophils, lymphatic system, adaptive immunity, auto‐immunity, chronic inflammation, cancer

## Abstract

Recent advances have provided evidence for the involvement of neutrophils in both innate and adaptive immunity, robustly challenging the old dogma that neutrophils are short‐lived prototypical innate immune cells solely involved in acute responses to microbes and exerting collateral tissue damage. There is now ample evidence showing that neutrophils can migrate into different compartments of the lymphoid system where they contribute to the orchestration of the activation and/or suppression of lymphocyte effector functions in homeostasis and during chronic inflammation, such as autoimmune disorders and cancer. In support of this notion, neutrophils can generate a wide range of cytokines and other mediators capable of regulating the survival, proliferation and functions of both T and B cells. In addition, neutrophils can directly engage with lymphocytes and promote antigen presentation. Furthermore, there is emerging evidence of the existence of distinct and diverse neutrophil phenotypes with immunomodulatory functions that characterise different pathological conditions, including chronic and autoimmune inflammatory conditions. The aim of this review is to discuss the mechanisms implicated in neutrophil trafficking into the lymphoid system and to provide an overview of the immuno‐regulatory functions of neutrophils in health and disease in the context of adaptive immunity. Copyright © 2018 The Authors. *The Journal of Pathology* published by John Wiley & Sons Ltd on behalf of Pathological Society of Great Britain and Ireland.

## Introduction

Neutrophils are short‐lived immune cells that are commonly accepted as being the first leucocyte sub‐type to be recruited in large numbers from the blood circulation into inflamed tissues. Their migration through the cellular and matrix components of blood vessel walls is a tightly regulated process involving intricate cellular and molecular interactions, as summarised in recent articles 1, 2, 3, 4, 5, 6. This phenomenon is fundamental for an effective innate immune response against infections or acute injury, providing a host defence mechanism that clears tissues of invading pathogens, dead cells and potentially harmful debris. Neutrophil diapedesis also plays an important role in regulating the phenotype and activation state of emigrated neutrophils at sites of inflammation 7. Key effector functions of neutrophils include phagocytosis and killing of intracellular or extracellular pathogens, tissue remodelling and secretion of chemotactic and immunomodulatory molecules that can further regulate the recruitment and activation of other pro‐inflammatory leucocytes 8, 9, 10, 11. To facilitate these functions, activated neutrophils can also release a wide range of granular proteases and cytotoxic factors, as well as generate a host of reactive oxygen species. Whilst such capabilities are essential armoury for destroying microorganisms, they can also cause collateral damage to host tissues, rendering clearance of apoptotic neutrophils an essential component of resolving an inflammatory response 12, 13. Another form of cell death program for neutrophils is via NETosis, a dynamic process associated with the generation of neutrophil extracellular traps (NETs) 14, 15, 16. NETs are generated through the release of decondensed chromatin and granular enzymes into the extracellular space surrounding leucocytes as means of controlling the dissemination of infectious microorganisms 14, 17, 18. Whilst the role of NETs in the direct killing of pathogens remains contentious 14, 19, 20, there is strong evidence to indicate that the formation of NETs *in vivo* can be detrimental to the host. Pathological induction of NETs, such as that induced under conditions of sterile injury (e.g. ischemia/reperfusion injury) can also cause tissue damage and indeed NETosis has been implicated to the pathogenesis of a wide range of non‐infectious inflammatory disorders 21, 22, 23, 24. Collectively, excessive recruitment, activation and/or inefficient clearance of infiltrated neutrophils is now categorically associated with the development of numerous acute pathological conditions such as myocardial infarction, stroke and tissue damage caused by ischemic insults 22, 25, 26 and there is now a growing interest in the pathogenic potential of neutrophils in chronic conditions such as cancer 27, 28, 29. Furthermore, the association of neutrophils with multiple autoimmune disorders (e.g. RA, lupus, multiple sclerosis, Crohn's disease and vasculitis) 21, 30, 31, 32, 33, 34 has invigorated the interest in neutrophils as potential players in regulation of adaptive immunity.

Nearly 50 years ago, whilst studying the trafficking of immune cells in sheep, Smith and colleagues discovered that the neutrophil ‘endgame’ was not limited to apoptosis within inflamed tissues but that these cells could be detected in the peripheral lymph of the animals 35. The authors speculated that this response provided a means for neutrophils to recirculate back into the blood, as opposed to contributing to adaptive immune responses taking place in the draining LNs. This hypothesis was upheld for a long time due to the difficulty in culturing neutrophils for prolonged periods *in vitro* and the general and well‐accepted assumption that the neutrophil life expectancy in the blood circulation did not exceed 1 day. However, advancements in techniques for tracking neutrophils *in vivo*, and the overall better understanding of neutrophil biology, have unequivocally demonstrated that neutrophils can exhibit prolonged survival both *in vitro* and *in vivo* 36. For example, whilst human neutrophils are reported to exhibit a blood circulation period of up to 5 days 37, cytokines such as GM‐CSF 38, 39, bacteria‐derived products 40, hypoxic conditions 41 and diapedesis through blood endothelium 42, 43 can protect neutrophils from rapid cell death. Such findings have led to greater acceptance of neutrophils as regulators of the adaptive immunity as supported by a large body of evidence demonstrating that neutrophils can migrate into secondary lymphoid organs such as LNs during bacterial and parasitic infections as well as during vaccination challenge protocols 44, 45, 46, 47. These works also reported on the ability of neutrophils to secrete numerous immunomodulatory molecules affecting lymphocytes 48, 49, 50 as well as to directly interact with lymphocytes acting as antigen presenting cells (APCs) 51, 52, 53, 54, 55, 56. Collectively there is now a renewed interest in models related to neutrophil trafficking into the lymphoid system and the pathophysiological consequences of this response, concepts that are reviewed below.

## Neutrophil trafficking to lymphoid tissues

Neutrophil migration to lymphoid organs was first demonstrated in animal models through localising neutrophils within the draining LNs of tissues infected with microorganisms or following vaccine challenge 44, 45, 46, 47, 57. The development and use of advanced imaging tools, such as intravital confocal microscopy, in conjunction with genetically modified animals exhibiting fluorescently tagged neutrophils (e.g. *LysM‐GFP‐ki* mice), enabled detailed analysis of the dynamics of neutrophil–lymphatic vessel interactions as well as the role of specific molecular cues involved in this process (Table 1). These studies provided direct evidence for neutrophils migrating to LNs via afferent lymphatics present in inflamed tissues. Interestingly, this trafficking response was rapid (within 6–12 h post insult) and transient as very few neutrophils could be detected in the LNs past 48 h 46, 57. The first molecular pathway linked with this response involved CCR7 and its cognate ligands CCL21 and CCL19 45. Importantly, the work of Beauvillain and colleagues demonstrated the presence of intracellular stores (possibly secretory vesicles) of CCR7 in both human and murine neutrophils isolated from the blood and bone marrow, respectively. Interestingly, whilst CCR7 was almost undetectable on the cell surface of neutrophils, the introduction of a purification step to enrich the neutrophil population *in vitro* enabled the detection of the molecule on the membrane. These findings suggested that priming of leucocytes was essential for the trafficking of CCR7 from intracellular stores to the cell membrane. Indeed, stimulation of human neutrophils with the cytokine GM‐CSF could promote their migration towards a CCL21/CCL19 chemotactic gradient *in vitro*, a response that was potentiated by LPS or IL17. *In vivo*, we and others have demonstrated that upon immunisation with complete Freund's adjuvant (CFA), CCR7 deficient mice have reduced numbers of neutrophils migrating into tissue‐associated lymphatic capillaries and into draining LNs, as compared to wild‐type control littermates 45, 57. Our study also provided evidence that immunisation of the animals with CFA induced the local release of endogenous TNF, a response essential for the control of neutrophil entry into lymphatic capillaries in a CCR7‐dependent manner. Furthermore, the trafficking of CCR7‐deficient neutrophils through afferent lymphatic vessels was completely suppressed in TNF‐induced inflammation 57. Interestingly, significant CCR7 expression was detected on the cell surface of tissue‐infiltrated neutrophils but not on cells from the blood circulation, or on tissue‐infiltrated neutrophils deficient in TNF receptors (both TNFRI and TNFRII). Collectively, these findings provide compelling evidence to indicate the necessity of priming for neutrophil migration into the lymphoid system and identify tissue‐derived TNF as a key modulator of *in vivo* expression and function of CCR7 on neutrophils. Other studies have suggested that the CXCR4/CXCL12 axis is critical for neutrophil entry into the lymphatic system 53, 59. CXCR4 is a chemokine receptor expressed at low levels on the surface of mature healthy neutrophils; but this molecule is upregulated on the membrane of aged neutrophils, a response associated with the egress of senescent neutrophils from the circulation 61, 62, 63. In a study using a murine model of *Staphylococcus aureus* infection, a specific inhibitor of CXCR4, AMD3100, was shown to significantly reduce the migration of neutrophils into afferent lymphatic vessels and draining LNs, whilst CCR7‐deficient neutrophils exhibited normal trafficking to the lymphatic system 53. Similar results were obtained in a mouse model of immunisation associated with pre‐activation of neutrophils with immune complexes 59. Differential involvement of distinct chemokine axes in regulating neutrophil entry into the lymphatics might depend on the inflammatory models used, the degree of activation of neutrophils or the potential existence of yet not described tissue‐specific mechanisms. Another chemokine implicated in human neutrophil migration into the lymphatic system is the prototypical neutrophil chemoattractant CXCL8. A study by Rigby and colleagues recently demonstrated that human dermal lymphatic endothelial cells (LECs) can secrete this chemokine and promote the migration of human neutrophils through LEC monolayers *in vitro* 60. Similarly, isolated LECs from murine skin exhibited enhanced gene expression of CXCL1 (a homologue of human CXCL8) upon stimulation 64. However, blockade of CXCL1 protein, or its receptor CXCR2, had no effect on murine neutrophil recruitment to lymphatic vessels *in vivo* 53, 57, highlighting potential discrepancies between putative *in vivo* and *in vitro* – and species – scenarios. In addition to chemokines, adhesion molecules such as ICAM‐1 and VCAM‐1 have been reported to be expressed by stimulated LECs and to support human and mouse neutrophil–lymphatic vessel interactions via binding to leucocyte β2 integrins (e.g. Mac‐1) both *in vitro* and *in vivo* 53, 57, 60. For instance, Mac‐1/ICAM‐1 interaction is critical for the attachment and crawling of murine neutrophils along the luminal aspect of lymphatic endothelium *in vivo* 57. Similarly, Mac‐1 blockade inhibited the entry of neutrophils into lymphatics of mouse skin that had been locally injected with bacteria 53 whilst another neutrophil‐expressed integrin, LFA‐1, is apparently dispensable for neutrophil intravasation into lymphatic vessels 59.

**Table 1:** Molecules implicated to neutrophil migration into the lymphoid system

Molecule	Surface expression	Role in entry via lymphatic capillaries	Role in entry via HEVs	Inflammatory model (when relevant)	References
CCR7	• Low neutrophil surface expression necessitating priming: → *In vitro* (GM‐CSF/IL‐17/LPS* or TNF†) → *In vivo* (post‐extravasation, TNF‐dependent†) • Expressed on tumour‐associated neutrophil N1 type*	Yes but depends on the nature of the reaction	No	• *In vitro* stimulation* • Immunisation (CFA)† • TNF‐induced inflammation† • Early lung tumour*	45, 53, 57, 58
CXCR4	• Low surface level on mature neutrophils • High surface level on aged neutrophils	Yes (but depends on the nature of the reaction)	Yes	• *S. aureus* infection† • Immunisation with immuno‐complex activation† • Immunisation (CFA)†	53, 57, 59
CXCR2	High levels on mature naïve neutrophils	Yes, *in vitro* No, *in vivo*	*In vivo* during PDT	• *In vitro* stimulation of dermal LECs* • *S. aureus* infection† • Immunisation (CFA)† • PDT in cancer†	53, 57, 58, 60
CD54 (ICAM‐1)	Up‐regulation on LVs and LECs upon inflammation (TNF dependent)	Yes (promote adhesion and luminal crawling)	Yes	• *S. aureu*s infection† • Immunisation, TNF‐induced inflammation† • Immunisation with immuno‐complex activation† • *In vitro* stimulation of dermal LECs*	53, 57, 59, 60
CD11b (Mac‐1)	High expression on all neutrophils	Yes (promote adhesion and luminal crawling)	Yes	• *S. aureus* infection† • Immunisation, TNF‐induced inflammation† • Immunisation with immuno‐complex activation† • *In vitro* stimulation of dermal LECs*	53, 57, 59, 60
CD11a (LFA1)	Expressed on all neutrophils	Rarely	Yes	• *S. aureus* infection† • Immunisation with immuno‐complex activation†	53, 59
CD62L (L‐selectin)	Expressed on neutrophils, shed upon stimulation/extravasation	No	Yes	• *S. aureus* infection† • Immunisation with immuno‐complex activation† • PDT in cancer†	53, 58, 59
CD62P (P‐selectin)	Endothelial selectin, upregulated upon stimulation of ECs	Not tested	Yes	• Immunisation with immuno‐complex activation†	59
CD62E (E‐selectin)	Endothelial selectin, upregulated upon stimulation of ECs	*In vitro* only	Yes	• *In vitro* stimulation of dermal LECs*	59
CD168 (PSGL‐1)	Selectin ligand expressed by both LECs and neutrophils	No	Yes	• *S. aureus* infection† • Immunisation with immuno‐complex activation†	53, 59
PNAd	Expressed on HEVs	No	Yes	• *S. aureus* infection† • Immunisation with immuno‐complex activation†	53, 59

* Shown in humans.

† Shown in mouse models.

Several studies have also demonstrated the capacity of blood circulating neutrophils to enter LNs via high endothelial venules (HEVs) during infection, post immune complex activation and antigen sensitisation 53, 59, 65. To date the only chemotactic axis described to be important for the migration of neutrophils through HEVs is CXCR4/CXCL12 axis, whilst a role for CCR7 and its cognate ligands CCL21/CCL19 have been completely ruled out 53, 59. Other molecules associated with neutrophil–HEV interactions are P‐ and L‐selectins and their cognate ligand PSGL‐1 as well as endothelial cell ICAM‐1 and leucocyte integrins Mac‐1 and LFA‐1 53, 59.

Collectively, there is now ample evidence to demonstrate the capacity of neutrophils to migrate during inflammation into LNs via two distinct routes, though the molecular pathways of such events require further exploration. Nevertheless, the fact that LN neutrophils can originate from either the blood circulation or inflamed tissues suggests potential differential modes of neutrophil‐mediated regulation of the adaptive immunity. The following section discusses the immuno‐modulatory functions of neutrophils in the context of lymphocyte activation.

## Neutrophil regulation of lymphocyte functions

Recent advances in neutrophil biology, including studies detailed in the previous section, have acknowledged these cells as key players at the interface of innate and adaptive immunity in both physiological homeostasis and pathological inflammation. The rapid and transient nature of their trafficking to LNs, with a dwelling time in these organs not exceeding 2–7 days following an initial inflammatory insult 53, 65, has led to the hypothesis that neutrophils can facilitate the transport of pathogens and antigens into LNs. It is considered that the latter are subsequently transferred to resident DCs and macrophages already present in secondary lymphoid organs through engulfment of neutrophil‐derived vesicles (e.g. apobodies, exosomes or micro‐vesicles). Furthermore, it is envisaged that through this cascade of events macrophages and conventional APCs such as DCs, can process and present exogenous antigens to lymphocytes 46, 47. This concept is supported by the detection of apoptotic neutrophils within LNs of *S. aureus* infected animals 53. In addition to this notion, it is now clear that activated neutrophils can secrete numerous immune modulatory molecules that can directly stimulate the recruitment, activation and functions of lymphocytes 48, 49, 50. More importantly, neutrophils can impact the regulatory functions of lymphocytes via direct neutrophil–lymphocyte interactions and antigen presentation 66. For example, several studies have demonstrated the existence of the so‐called neutrophil‐DC hybrids, an activated neutrophil sub‐type that exhibit characteristics of DCs (e.g. expressing MHC‐II CD80 and CD86) and capable of presenting exogenous antigen to both CD4+ and CD8+ T lymphocytes *in vivo* and *in vitro* 51, 67, 68, 69. This phenomenon was confirmed by confocal imaging that showed the dynamics of cell contacts between neutrophils and lymphocytes in mouse models of infection and immunisation 46. In humans, neutrophils can also stimulate the antigen‐specific proliferation of both naïve and memory T cells through MHC‐II expression 70. Furthermore, neutrophils can positively and directly modulate B cell activation, survival and differentiation by means of secretion of cytokines such as B‐cell‐Activating Factor of the tumour necrosis factor family (BAFF) 71, and a proliferation‐inducing ligand (APRIL) 72. Whilst neutrophils are not usually seen in the germinal centres of LNs, a sub‐population has been found in the peri‐follicular areas and marginal zone of the spleen in both humans and mice 73, 74. These cells, termed B‐helper neutrophils, have been reported to induce T‐cell independent production of IgG and IgA (following immunoglobulin class switching) via the production of large amount of BAFF, APRIL, CD40L and interleukin‐21. B helper neutrophils may therefore represent a central mechanism for enhancing antibody production and effective humoral responses in a T‐cell independent manner 75, 76, 77, 78. Direct interactions between neutrophils and B cells have also been observed in real time in LNs of *S. aureus* infected animals 65.

In recent years, the homogeneity of neutrophil population has become increasingly questionable, with detection of neutrophils exhibiting distinct functions and profiles leading to the concept of different subsets 79, 80, 81, 82. Several studies have indeed demonstrated that alternatively activated and/or HEV‐recruited neutrophils can regulate the adaptive immune response by inhibiting B and T lymphocyte responses, in particular in the context of antibody production during vaccination challenge and infections 46, 53, 65, 83, 84. Whilst the mechanisms associated with such functions are not fully understood, neutrophil suppressive properties on T lymphocyte activities involve the release of reactive oxygen species, NO or Arginase 1 (Arg‐1) in close vicinity of targeted lymphocytes 85, 86. Neutrophils can also directly inhibit T cell functions through cell–cell interactions in a Mac‐1 or PD‐L1 dependent manner during HIV infections, systemic endotoxemia and cancer 87, 88, 89, 90. Furthermore, whilst neutrophil depletion can increase the production of antigen‐specific IgG and IgM, during *S. aureus* infection, B‐helper neutrophils were shown to limit the production of IgM through release of TGF‐β1 65. Finally, recent studies have also investigated the role of specific sub‐types of neutrophils in the context of Treg expansion and recruitment. For instance, an elegant study by Nadkarni and colleagues has demonstrated that neutrophils sensitised to pregnancy hormones promote the differentiation of a unique population of FOXP3+ CD4+ Tregs with a specific secretory phenotype (release of IL‐10, VEGF and IL‐17) via the transfer of neutrophil‐derived proteins such as forkhead box protein‐1 to naive T cells 91.

Taken together, there is now unquestionable evidence supporting neutrophil trafficking into the lymphatic system and the ability of these cells to regulate lymphocyte functions. These works have promoted more interest into the potential role of neutrophils in chronic disorders, including autoimmune diseases, as discussed below.

## Diversity of neutrophil phenotype and pathogenic potential in chronic and autoimmune diseases

**Figure 1:**
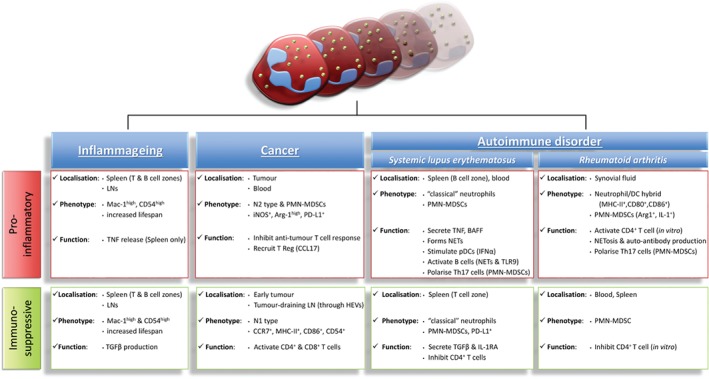
Neutrophils and the regulation of the adaptive immune response in diseases. Neutrophils have been implicated in the regulation of T and B cell activities in many pathological scenarios including inflammageing, cancer and autoimmune disorders such as RA and SLE. Neutrophils can both stimulate and immunosuppress lymphocyte functions through various mechanisms due to the plasticity of their phenotype, localisation and environmental priming. For instance they can secrete activating cytokines or act as APCs to stimulate lymphocyte proliferation and auto‐antibody production (e.g. via NETosis). In contrast, other sub‐types of neutrophils have been shown to directly inhibit T cell activation through the release of TGFβ, NO and Arg‐1 as well as through the expression and direct engagement of PD‐L1 with T cells.

The notable and now widely accepted diversity of neutrophil functions has placed a spotlight on the potential existence of distinct neutrophil‐subsets and their association with a broad range of pathologies (see examples in Figure 1). This includes sub‐types of neutrophils that can exert stimulatory and suppressive effects on lymphocyte functions and the potential association of these cells to chronic disorders such as cancer, and autoimmune diseases, such as SLE or RA 81, 92, 93. One example relates to granulocytic myeloid‐derived suppressor cells (PMN‐MDSCs), detected in cancer patients, considered by some researchers as a subset of neutrophils 81, 86, 94, 95. This section reviews recent findings associated with neutrophil trafficking to lymphoid tissues in cancer and chronic inflammatory and autoimmune pathologies.

### The duality of neutrophil functions in cancer

In human cancer, tumour‐associated neutrophils have been classified as having anti‐tumour (N1) and pro‐tumour (N2) properties  92, 96. There is also a body of evidence supporting the presence of increased numbers of PMN‐MDSCs in blood, within the tumour microenvironment and peripheral lymphoid organs (tumour‐draining LNs and spleen) in this pathology in humans and mice 97. The dominant view is that neutrophils and PMN‐MDSCs are important contributors to tumour progression through their ability to promote angiogenesis, proliferation and metastasis of cancer cells 94, 95, 98, as supported by experimental murine models of cancer 99. Furthermore, N2 neutrophils and PMN‐MDSCs have been shown to exhibit immunosuppressive properties via PD‐L1‐dependent immunosuppression of Th1 cell proliferation 89 and to promote the expansion and recruitment of regulatory T lymphocytes via the secretion of CCL17 100, 101, 102. Of note, a distinct subpopulation of human neutrophils (exhibiting neutrophil‐DC hybrid characteristics) has been discovered within the tumour‐microenvironment and draining LNs during the early stage of lung cancer 103, 104. These tumour‐associated neutrophils were reported to induce efficient anti‐tumour responses from memory CD8+ and CD4+ T cells *in vitro* and expressed CCR7 at their surface 105. In support of these findings, a recent study has described the presence of CXCR2^+^ neutrophils exhibiting IL‐6 secretory pro‐inflammatory phenotype in the draining LN of gastric tumours 106. At present, the literature lacks further detailed information regarding the route, mechanisms of migration and activity of such distinct populations of neutrophils during the development of the pathology *in vivo*. Yet, in the context of anti‐tumour therapy, using a mouse model of colon carcinoma, Brackett and colleagues demonstrated that photodynamic therapy (PDT) was also associated with the rapid recruitment of neutrophils (i.e. within 4 h post PDT) into tumour‐draining LNs through HEVs in an IL‐17 and CXCR2/CXCL2 (but not CXCL1)‐dependent manner 58, 107.The authors also showed the importance of L‐selectin and of peripheral node addressin (PNAd) in neutrophil entry through HEVs in this model. Functionally, PDT has been associated with the development of neutrophil‐dependent and tumour‐specific primary and memory CD8(+) T cell responses through the direct induction of T lymphocyte proliferation and/or survival 108.

### Neutrophils in chronic inflammatory and auto‐immune disorders

Neutrophils with immuno‐modulatory activities have also been detected in ageing, now regarded by some researchers as a low grade chronic inflammatory state (i.e. inflammageing). Accordingly, in aged mice neutrophils have been detected in high numbers in both T‐cell and B‐cell areas of the secondary lymphoid organs (i.e. LNs and spleen) 109. The increased neutrophil trafficking to lymphoid organs was associated with enhanced life‐span of the infiltrated neutrophils and their altered phenotype. Specifically, in healthy aged mice, lymphoid tissue neutrophils expressed an activated phenotype characterised by high levels of Mac‐1 and ICAM‐1 and the concomitant synthesis of both pro‐inflammatory and anti‐inflammatory cytokines TNF and TGFβ, respectively. At present it is unclear what the role of these neutrophils is, but it is speculated that the atypical phenotype of lymphoid organ neutrophils could contribute to ageing‐associated dysregulation of normal adaptive immune responses, e.g. in infections 109.

In autoimmune pathologies such as RA, human synovial neutrophils express transcripts and proteins of MHC‐II and co‐stimulatory molecules CD80 and CD86 during the early phase of the disease 110, 111. *In vitro*, freshly isolated synovial neutrophils are capable of stimulating a proliferative response in T‐helper cells 111. Whilst rather intriguing, such seminal studies did not demonstrate where (e.g. tissue localisation) and when (e.g. phase of the disorder) during RA such neutrophil responses were functionally relevant to disease progression. Indeed, it would be important to elucidate if neutrophils can regulate T cell functions in RA *in vivo* within the secondary lymphoid organs. Related to this caveat, using a murine model of lupus, Bird and colleagues demonstrated that neutrophils can preferentially form close interactions with T cells in the early phase of the disease, whilst in the advanced stage, they mainly accumulate in B cell areas of the spleen, reminiscent of a B‐helper phenotype 112. Furthermore, transcriptome analysis demonstrated that neutrophils exhibited high expression of PD‐L1, TGFβ and IL‐1RA during the early disease phase. The immunosuppressive activity of neutrophils during this period was confirmed by the negative effect of neutrophil depletion on disease progression, germinal centre formation and production of anti‐double strand DNA antibodies. Conversely, during the late phase, neutrophils enriched in splenic B‐cell regions increased their expression of TNF and BAFF, indicating that they may contribute to the expansion of auto‐reactive B lymphocytes during disease progression 112. Interestingly, B‐helper neutrophils have been shown to be more susceptible to NETosis 74 and hence it has been suggested that, due to their localisation and specific phenotypic characteristics, these cells may promote the expansion and survival of auto‐reactive B cells. A role for B‐helper neutrophils in supporting adaptive autoimmune responses has indeed been reported in many autoimmune disorders, including, RA, SLE and in anti‐neutrophil cytoplasmic antibody‐associated (ANCA) vasculitis 73. The mechanism of action of these neutrophils is not completely understood but their enhanced capacity to generate NETs was suggested to provide an abundant source of auto‐antigens characteristic of these pathologies 17, 113, 114, 115. Supporting this concept, a recent study by Gestermann and colleagues demonstrated that NET components could directly activate memory B cells through TLR9 stimulation, leading to the production of pathogenic autoantibodies in a T cell‐independent manner 116. Moreover, NETs can also stimulate plasmacytoid dendritic cells to secrete interferon‐α which in turn, promotes NETosis of neutrophils. This cascade perpetuates a vicious cycle that can be observed in pathologies such as vasculitis, SLE, psoriasis and type‐1 diabetes 117, 118, 119, 120.

The number of PMN‐MDSCs has also been reported to increase in RA and SLE patients 121, 122, 123 as well as in several experimental animal models of lupus 124, collagen‐induced arthritis 125, EAE 126, type‐1 diabetes and inflammatory bowel disease 127, 128. Whilst many studies have correlated MDSCs with disease severity, their role in regulating lymphocyte responses remains controversial 129. Indeed, whilst in RA, SLE and EAE, PMN‐MDSCs have been shown to inhibit both proliferation of T helper‐cells and their production of cytokines (e.g. interferon‐γ and IL‐2) in an Arg‐1‐dependent manner, these cells also produce pro‐inflammatory cytokines (e.g. IL‐1 and TNF). The latter can promote the differentiation of naïve T lymphocytes into Th17 cells, suggesting that in the disease inflammatory context, PMN‐MDSCs can exhibit a pathogenic phenotype 122, 130, 131.

Overall, whilst the current literature supports the concept of neutrophils orchestrating a pathogenic response during autoimmune and chronic inflammatory disorders, further investigations are required to determine if and how neutrophil trafficking into lymphoid organs might be contributing to the aetiology of these diseases.

## Conclusion and perspectives

In addition to their essential role in protecting the host against acute harmful insults, recent studies have identified a broader role for neutrophils in both physiological and pathological immunity, and hence the development of numerous chronic and autoimmune inflammatory disorders. Specifically, it is now clear that, despite being short‐lived as compared to other immune cells, neutrophils can traffic into lymphoid tissues and contribute to shaping key adaptive immune responses. As such, current evidence supports the notion that in addition to dendritic cells and macrophages, neutrophils can act as a cellular bridge between innate and adaptive immunity in both health and disease. Whilst our understanding of the mechanisms of neutrophil trafficking and functions within lymphoid tissues is incomplete, it is now accepted that depending on the nature, duration or the site of the insult, neutrophils can enter the lymphatic system by using both afferent lymphatic vessels as well as venular portals within LNs. Once in secondary lymphoid organs, neutrophils can act as immune‐modulatory cells through cytokine production and via direct cell–cell interaction with other immune cells. Accordingly, neutrophils can orchestrate elaborate cellular and humoral responses as well as exert regulatory effects on lymphocyte functions. The development of new technologies applied to investigate the dynamics of neutrophil behaviour, phenotype and trafficking in both patient samples *ex vivo* and in animal models of inflammation *in vivo* has led to the identification of different sub‐types of neutrophils with distinct regulatory functions in health and disease. It is still debated whether neutrophil subsets might represent an acquired phenotype, and/or level of activation through environmental molecular cues, or are in fact ontogenically separate cell populations. Irrespective of this, renewed interest in this phenotypic diversity and associated varied effector functions, in conjunction with better understanding of the spatiotemporal localisation of neutrophils is opening challenging research opportunities. In particular, such works could pave the way towards addressing outstanding questions regarding the diverse functions of neutrophils during the development of autoimmune pathologies. Most importantly, association of different neutrophil sub‐types with the pathogenesis of defined disorders has the potential for stratifying patients in terms of disease severity as well as identify new therapeutic targets in chronic and autoimmune conditions and cancer.

## Author contributions statement

MBV performed the literature search and wrote the manuscript. SN contributed to the writing and revision of the manuscript. Both authors approved the final version.

## References

[1] Muller WA . Transendothelial migration: unifying principles from the endothelial perspective. Immunol Rev 2016; 273: 61–75. 27558328 10.1111/imr.12443 PMC5090979

[2] Nourshargh S , Alon R . Leukocyte migration into inflamed tissues. Immunity 2014; 41: 694–707. 25517612 10.1016/j.immuni.2014.10.008

[3] Reglero‐Real N , Colom B , Bodkin JV , *et al* Endothelial cell junctional adhesion molecules: role and regulation of expression in inflammation. Arterioscler Thromb Vasc Biol 2016; 36: 2048–2057. 27515379 10.1161/ATVBAHA.116.307610 PMC5035539

[4] Schnoor M , Alcaide P , Voisin MB , *et al* Crossing the vascular wall: common and unique mechanisms exploited by different leukocyte subsets during extravasation. Mediators Inflamm 2015; 2015: 946509. 26568666 10.1155/2015/946509 PMC4629053

[5] Vestweber D . How leukocytes cross the vascular endothelium. Nat Rev Immunol 2015; 15: 692–704. 26471775 10.1038/nri3908

[6] Voisin MB , Nourshargh S . Neutrophil transmigration: emergence of an adhesive cascade within venular walls. J Innate Immun 2013; 5: 336–347. 23466407 10.1159/000346659 PMC6741604

[7] Nourshargh S , Marelli‐Berg FM . Transmigration through venular walls: a key regulator of leukocyte phenotype and function. Trends Immunol 2005; 26: 157–165. 15745858 10.1016/j.it.2005.01.006

[8] Lerman YV , Kim M . Neutrophil migration under normal and sepsis conditions. Cardiovasc Hematol Disord Drug Targets 2015; 15: 19–28. 25567338 10.2174/1871529x15666150108113236 PMC5111082

[9] Soehnlein O , Lindbom L , Weber C . Mechanisms underlying neutrophil‐mediated monocyte recruitment. Blood 2009; 114: 4613–4623. 19696199 10.1182/blood-2009-06-221630

[10] Soehnlein O , Zernecke A , Eriksson EE , *et al* Neutrophil secretion products pave the way for inflammatory monocytes. Blood 2008; 112: 1461–1471. 18490516 10.1182/blood-2008-02-139634 PMC3400540

[11] Theilgaard‐Monch K , Porse BT , Borregaard N . Systems biology of neutrophil differentiation and immune response. Curr Opin Immunol 2006; 18: 54–60. 16343884 10.1016/j.coi.2005.11.010

[12] Greenlee‐Wacker MC . Clearance of apoptotic neutrophils and resolution of inflammation. Immunol Rev 2016; 273: 357–370. 27558346 10.1111/imr.12453 PMC5000862

[13] Ortega‐Gomez A , Perretti M , Soehnlein O . Resolution of inflammation: an integrated view. EMBO Mol Med 2013; 5: 661–674. 23592557 10.1002/emmm.201202382 PMC3662311

[14] Brinkmann V , Reichard U , Goosmann C , *et al* Neutrophil extracellular traps kill bacteria. Science 2004; 303: 1532–1535. 15001782 10.1126/science.1092385

[15] Brinkmann V , Zychlinsky A . Beneficial suicide: why neutrophils die to make NETs. Nat Rev Microbiol 2007; 5: 577–582. 17632569 10.1038/nrmicro1710

[16] Fuchs TA , Abed U , Goosmann C , *et al* Novel cell death program leads to neutrophil extracellular traps. J Cell Biol 2007; 176: 231–241. 17210947 10.1083/jcb.200606027 PMC2063942

[17] Amulic B , Hayes G . Neutrophil extracellular traps. Curr Biol 2011; 21: R297–R298. 21549944 10.1016/j.cub.2011.03.021

[18] Ermert D , Urban CF , Laube B , *et al* Mouse neutrophil extracellular traps in microbial infections. J Innate Immun 2009; 1: 181–193. 20375576 10.1159/000205281 PMC6951040

[19] Menegazzi R , Decleva E , Dri P . Killing by neutrophil extracellular traps: fact or folklore? Blood 2012; 119: 1214–1216. 22210873 10.1182/blood-2011-07-364604

[20] Malachowa N , Kobayashi SD , Quinn MT , *et al* NET confusion. Front Immunol 2016; 7: 259. 27446089 10.3389/fimmu.2016.00259 PMC4923183

[21] Amulic B , Cazalet C , Hayes GL , *et al* Neutrophil function: from mechanisms to disease. Annu Rev Immunol 2012; 30: 459–489. 22224774 10.1146/annurev-immunol-020711-074942

[22] Jorch SK , Kubes P . An emerging role for neutrophil extracellular traps in noninfectious disease. Nat Med 2017; 23: 279–287. 28267716 10.1038/nm.4294

[23] Law SM , Gray RD . Neutrophil extracellular traps and the dysfunctional innate immune response of cystic fibrosis lung disease: a review. J Inflamm (Lond) 2017; 14: 29. 29299029 10.1186/s12950-017-0176-1 PMC5745605

[24] Soderberg D , Segelmark M . Neutrophil extracellular traps in vasculitis, friend or foe? Curr Opin Rheumatol 2018; 30: 16–23. 28957962 10.1097/BOR.0000000000000450

[25] Carbone F , Nencioni A , Mach F , *et al* Pathophysiological role of neutrophils in acute myocardial infarction. Thromb Haemost 2013; 110: 501–514. 23740239 10.1160/TH13-03-0211

[26] Perez‐de‐Puig I , Miro‐Mur F , Ferrer‐Ferrer M , *et al* Neutrophil recruitment to the brain in mouse and human ischemic stroke. Acta Neuropathol 2015; 129: 239–257. 25548073 10.1007/s00401-014-1381-0

[27] Ocana A , Nieto‐Jimenez C , Pandiella A , *et al* Neutrophils in cancer: prognostic role and therapeutic strategies. Mol Cancer 2017; 16: 137. 28810877 10.1186/s12943-017-0707-7 PMC5558711

[28] Sagiv JY , Michaeli J , Assi S , *et al* Phenotypic diversity and plasticity in circulating neutrophil subpopulations in cancer. Cell Rep 2015; 10: 562–573. 25620698 10.1016/j.celrep.2014.12.039

[29] Wang X , Qiu L , Li Z , *et al* Understanding the multifaceted role of neutrophils in cancer and autoimmune diseases. Front Immunol 2018; 9: 2456. 30473691 10.3389/fimmu.2018.02456 PMC6237929

[30] Cascao R , Rosario HS , Souto‐Carneiro MM , *et al* Neutrophils in rheumatoid arthritis: more than simple final effectors. Autoimmun Rev 2010; 9: 531–535. 20060506 10.1016/j.autrev.2009.12.013

[31] Segal AW . The role of neutrophils in the pathogenesis of Crohn's disease. Eur J Clin Invest 2018; 48(suppl 2): e12983. 29931668 10.1111/eci.12983

[32] Stephens M , Liao S . Neutrophil–lymphatic interactions during acute and chronic disease. Cell Tissue Res 2018; 371: 599–606. 29423716 10.1007/s00441-017-2779-5

[33] Swirski FK , Robbins CS . Neutrophils usher monocytes into sites of inflammation. Circ Res 2013; 112: 744–745. 23449542 10.1161/CIRCRESAHA.113.300867

[34] Woodberry T , Bouffler SE , Wilson AS , *et al* The emerging role of neutrophil granulocytes in multiple sclerosis. J Clin Forensic Med 2018; 7: E511. 10.3390/jcm7120511 PMC6306801 30513926

[35] Smith JB , McIntosh GH , Morris B . The traffic of cells through tissues: a study of peripheral lymph in sheep. J Anat 1970; 107: 87–100. 5473295 PMC1234166

[36] Tak T , Tesselaar K , Pillay J , *et al* What's your age again? Determination of human neutrophil half‐lives revisited. J Leukoc Biol 2013; 94: 595–601. 23625199 10.1189/jlb.1112571

[37] Pillay J , den Braber I , Vrisekoop N , *et al* In vivo labeling with 2H_2_O reveals a human neutrophil lifespan of 5.4 days. Blood 2010; 116: 625–627. 20410504 10.1182/blood-2010-01-259028

[38] Coxon A , Tang T , Mayadas TN . Cytokine‐activated endothelial cells delay neutrophil apoptosis in vitro and in vivo. A role for granulocyte/macrophage colony‐stimulating factor. J Exp Med 1999; 190: 923–934. 10510082 10.1084/jem.190.7.923 PMC2195653

[39] Kobayashi SD , Voyich JM , Whitney AR , *et al* Spontaneous neutrophil apoptosis and regulation of cell survival by granulocyte macrophage‐colony stimulating factor. J Leukoc Biol 2005; 78: 1408–1418. 16204629 10.1189/jlb.0605289

[40] Hachiya O , Takeda Y , Miyata H , *et al* Inhibition by bacterial lipopolysaccharide of spontaneous and TNF‐alpha‐induced human neutrophil apoptosis in vitro. Microbiol Immunol 1995; 39: 715–723. 8577286 10.1111/j.1348-0421.1995.tb03247.x

[41] Walmsley SR , Print C , Farahi N , *et al* Hypoxia‐induced neutrophil survival is mediated by HIF‐1alpha‐dependent NF‐kappaB activity. J Exp Med 2005; 201: 105–115. 15630139 10.1084/jem.20040624 PMC2212759

[42] McGettrick HM , Lord JM , Wang KQ , *et al* Chemokine‐ and adhesion‐dependent survival of neutrophils after transmigration through cytokine‐stimulated endothelium. J Leukoc Biol 2006; 79: 779–788. 16461737 10.1189/jlb.0605350 PMC3119451

[43] Seely AJ , Swartz DE , Giannias B , *et al* Reduction in neutrophil cell surface expression of tumor necrosis factor receptors but not Fas after transmigration: implications for the regulation of neutrophil apoptosis. Arch Surg 1998; 133: 1305–1310. 9865647 10.1001/archsurg.133.12.1305

[44] Abadie V , Badell E , Douillard P , *et al* Neutrophils rapidly migrate via lymphatics after *Mycobacterium bovis* BCG intradermal vaccination and shuttle live bacilli to the draining lymph nodes. Blood 2005; 106: 1843–1850. 15886329 10.1182/blood-2005-03-1281

[45] Beauvillain C , Cunin P , Doni A , *et al* CCR7 is involved in the migration of neutrophils to lymph nodes. Blood 2011; 117: 1196–1204. 21051556 10.1182/blood-2009-11-254490

[46] Chtanova T , Schaeffer M , Han SJ , *et al* Dynamics of neutrophil migration in lymph nodes during infection. Immunity 2008; 29: 487–496. 18718768 10.1016/j.immuni.2008.07.012 PMC2569002

[47] Maletto BA , Ropolo AS , Alignani DO , *et al* Presence of neutrophil‐bearing antigen in lymphoid organs of immune mice. Blood 2006; 108: 3094–3102. 16835380 10.1182/blood-2006-04-016659

[48] Costa S , Bevilacqua D , Cassatella MA , *et al* Recent advances on the crosstalk between neutrophils and B or T lymphocytes. Immunology 2019; 156: 23–32. 30259972 10.1111/imm.13005 PMC6283649

[49] Jaillon S , Galdiero MR , Del Prete D , *et al* Neutrophils in innate and adaptive immunity. Semin Immunopathol 2013; 35: 377–394. 23553214 10.1007/s00281-013-0374-8

[50] Mantovani A , Cassatella MA , Costantini C , *et al* Neutrophils in the activation and regulation of innate and adaptive immunity. Nat Rev Immunol 2011; 11: 519–531. 21785456 10.1038/nri3024

[51] Beauvillain C , Delneste Y , Scotet M , *et al* Neutrophils efficiently cross‐prime naive T cells in vivo. Blood 2007; 110: 2965–2973. 17562875 10.1182/blood-2006-12-063826

[52] Geng S , Matsushima H , Okamoto T , *et al* Emergence, origin, and function of neutrophil–dendritic cell hybrids in experimentally induced inflammatory lesions in mice. Blood 2013; 121: 1690–1700. 23305733 10.1182/blood-2012-07-445197 PMC3591794

[53] Hampton HR , Bailey J , Tomura M , *et al* Microbe‐dependent lymphatic migration of neutrophils modulates lymphocyte proliferation in lymph nodes. Nat Commun 2015; 6: 7139. 25972253 10.1038/ncomms8139 PMC4479041

[54] Matsushima H , Geng S , Lu R , *et al* Neutrophil differentiation into a unique hybrid population exhibiting dual phenotype and functionality of neutrophils and dendritic cells. Blood 2013; 121: 1677–1689. 23305731 10.1182/blood-2012-07-445189 PMC3591793

[55] Yao Y , Liu Y , Takashima A . Intravital imaging of neutrophil priming using IL‐1beta promoter‐driven DsRed reporter mice. J Vis Exp. 2016 Jun 22;(112). 10.3791/54070. PMC4993227 27403648

[56] Yao Y , Matsushima H , Ohtola JA , *et al* Neutrophil priming occurs in a sequential manner and can be visualized in living animals by monitoring IL‐1beta promoter activation. J Immunol 2015; 194: 1211–1224. 25527787 10.4049/jimmunol.1402018 PMC4297710

[57] Arokiasamy S , Zakian C , Dilliway J , *et al* Endogenous TNFalpha orchestrates the trafficking of neutrophils into and within lymphatic vessels during acute inflammation. Sci Rep 2017; 7: 44189. 28287124 10.1038/srep44189 PMC5347029

[58] Brackett CM , Muhitch JB , Evans SS , *et al* IL‐17 promotes neutrophil entry into tumor‐draining lymph nodes following induction of sterile inflammation. J Immunol 2013; 191: 4348–4357. 24026079 10.4049/jimmunol.1103621 PMC3795982

[59] Gorlino CV , Ranocchia RP , Harman MF , *et al* Neutrophils exhibit differential requirements for homing molecules in their lymphatic and blood trafficking into draining lymph nodes. J Immunol 2014; 193: 1966–1974. 25015824 10.4049/jimmunol.1301791

[60] Rigby DA , Ferguson DJ , Johnson LA , *et al* Neutrophils rapidly transit inflamed lymphatic vessel endothelium via integrin‐dependent proteolysis and lipoxin‐induced junctional retraction. J Leukoc Biol 2015; 98: 897–912. 26216937 10.1189/jlb.1HI0415-149R

[61] De Filippo K , Rankin SM . CXCR4, the master regulator of neutrophil trafficking in homeostasis and disease. Eur J Clin Invest 2018; 48(suppl 2): e12949. 29734477 10.1111/eci.12949 PMC6767022

[62] Furze RC , Rankin SM . Neutrophil mobilization and clearance in the bone marrow. Immunology 2008; 125: 281–288. 19128361 10.1111/j.1365-2567.2008.02950.x PMC2669132

[63] Rankin SM . The bone marrow: a site of neutrophil clearance. J Leukoc Biol 2010; 88: 241–251. 20483920 10.1189/jlb.0210112

[64] Vigl B , Aebischer D , Nitschke M , *et al* Tissue inflammation modulates gene expression of lymphatic endothelial cells and dendritic cell migration in a stimulus‐dependent manner. Blood 2011; 118: 205–215. 21596851 10.1182/blood-2010-12-326447

[65] Kamenyeva O , Boularan C , Kabat J , *et al* Neutrophil recruitment to lymph nodes limits local humoral response to Staphylococcus aureus . PLoS Pathog 2015; 11: e1004827. 25884622 10.1371/journal.ppat.1004827 PMC4401519

[66] Takashima A , Yao Y . Neutrophil plasticity: acquisition of phenotype and functionality of antigen‐presenting cell. J Leukoc Biol 2015; 98: 489–496. 25632045 10.1189/jlb.1MR1014-502R

[67] Blomgran R , Ernst JD . Lung neutrophils facilitate activation of naive antigen‐specific CD4+ T cells during *Mycobacterium tuberculosis* infection. J Immunol 2011; 186: 7110–7119. 21555529 10.4049/jimmunol.1100001 PMC3376160

[68] Iking‐Konert C , Ostendorf B , Sander O , *et al* Transdifferentiation of polymorphonuclear neutrophils to dendritic‐like cells at the site of inflammation in rheumatoid arthritis: evidence for activation by T cells. Ann Rheum Dis 2005; 64: 1436–1442. 15778239 10.1136/ard.2004.034132 PMC1755243

[69] Iking‐Konert C , Wagner C , Denefleh B , *et al* Up‐regulation of the dendritic cell marker CD83 on polymorphonuclear neutrophils (PMN): divergent expression in acute bacterial infections and chronic inflammatory disease. Clin Exp Immunol 2002; 130: 501–508. 12452842 10.1046/j.1365-2249.2002.02008.x PMC1906559

[70] Vono M , Lin A , Norrby‐Teglund A , *et al* Neutrophils acquire the capacity for antigen presentation to memory CD4(+) T cells in vitro and ex vivo. Blood 2017; 129: 1991–2001. 28143882 10.1182/blood-2016-10-744441 PMC5383872

[71] Scapini P , Bazzoni F , Cassatella MA . Regulation of B‐cell‐activating factor (BAFF)/B lymphocyte stimulator (BLyS) expression in human neutrophils. Immunol Lett 2008; 116: 1–6. 18155301 10.1016/j.imlet.2007.11.009

[72] Huard B , McKee T , Bosshard C , *et al* APRIL secreted by neutrophils binds to heparan sulfate proteoglycans to create plasma cell niches in human mucosa. J Clin Invest 2008; 118: 2887–2895. 18618015 10.1172/JCI33760 PMC2447926

[73] Cerutti A , Puga I , Magri G . The B cell helper side of neutrophils. J Leukoc Biol 2013; 94: 677–682. 23630389 10.1189/jlb.1112596 PMC3774846

[74] Puga I , Cols M , Barra CM , *et al* B cell‐helper neutrophils stimulate the diversification and production of immunoglobulin in the marginal zone of the spleen. Nat Immunol 2011; 13: 170–180. 22197976 10.1038/ni.2194 PMC3262910

[75] Deniset JF , Surewaard BG , Lee WY , *et al* Splenic Ly6G(high) mature and Ly6G(int) immature neutrophils contribute to eradication of *S. pneumoniae* . J Exp Med 2017; 214: 1333–1350. 28424248 10.1084/jem.20161621 PMC5413339

[76] Naranjo‐Gomez M , Lambour J , Piechaczyk M , *et al* Neutrophils are essential for induction of vaccine‐like effects by antiviral monoclonal antibody immunotherapies. JCI Insight 2018; 3: 97339. 29720574 10.1172/jci.insight.97339 PMC6012508

[77] Parsa R , Lund H , Georgoudaki AM , *et al* BAFF‐secreting neutrophils drive plasma cell responses during emergency granulopoiesis. J Exp Med 2016; 213: 1537–1553. 27432941 10.1084/jem.20150577 PMC4986521

[78] Scapini P , Cassatella MA . Location in the spleen dictates the function of murine neutrophils. J Exp Med 2017; 214: 1207–1209. 28424247 10.1084/jem.20170655 PMC5413343

[79] Beyrau M , Bodkin JV , Nourshargh S . Neutrophil heterogeneity in health and disease: a revitalized avenue in inflammation and immunity. Open Biol 2012; 2: 120134. 23226600 10.1098/rsob.120134 PMC3513838

[80] Deniset JF , Kubes P . Recent advances in understanding neutrophils. F1000Res 2016; 5: 2912. 28105328 10.12688/f1000research.9691.1 PMC5225409

[81] Deniset JF , Kubes P . Neutrophil heterogeneity: bona fide subsets or polarization states? J Leukoc Biol 2018; 103: 829–838. 29462505 10.1002/JLB.3RI0917-361R

[82] Kubes P . The enigmatic neutrophil: what we do not know. Cell Tissue Res 2018; 371: 399–406. 29404726 10.1007/s00441-018-2790-5

[83] Gorlino CV , Dave MN , Blas R , *et al* Association between levels of synovial anti‐citrullinated peptide antibodies and neutrophil response in patients with rheumatoid arthritis. Eur J Immunol 2018; 48: 1563–1572. 29879311 10.1002/eji.201847477

[84] Jee J , Bonnegarde‐Bernard A , Duverger A , *et al* Neutrophils negatively regulate induction of mucosal IgA responses after sublingual immunization. Mucosal Immunol 2015; 8: 735–745. 25563500 10.1038/mi.2014.105 PMC4481173

[85] Ingersoll SA , Laval J , Forrest OA , *et al* Mature cystic fibrosis airway neutrophils suppress T cell function: evidence for a role of arginase 1 but not programmed death‐ligand 1. J Immunol 2015; 194: 5520–5528. 25926674 10.4049/jimmunol.1500312 PMC4433848

[86] Pillay J , Tak T , Kamp VM , *et al* Immune suppression by neutrophils and granulocytic myeloid‐derived suppressor cells: similarities and differences. Cell Mol Life Sci 2013; 70: 3813–3827. 23423530 10.1007/s00018-013-1286-4 PMC3781313

[87] Bowers E , Scamurra RW , Asrani A , *et al* Decreased mutation frequencies among immunoglobulin G variable region genes during viremic HIV‐1 infection. PLoS One 2014; 9: e81913. 24409278 10.1371/journal.pone.0081913 PMC3883639

[88] de Kleijn S , Langereis JD , Leentjens J , *et al* IFN‐gamma‐stimulated neutrophils suppress lymphocyte proliferation through expression of PD‐L1. PLoS One 2013; 8: e72249. 24015224 10.1371/journal.pone.0072249 PMC3756078

[89] He G , Zhang H , Zhou J , *et al* Peritumoural neutrophils negatively regulate adaptive immunity via the PD‐L1/PD‐1 signalling pathway in hepatocellular carcinoma. J Exp Clin Cancer Res 2015; 34: 141. 26581194 10.1186/s13046-015-0256-0 PMC4652417

[90] Pillay J , Kamp VM , van Hoffen E , *et al* A subset of neutrophils in human systemic inflammation inhibits T cell responses through Mac‐1. J Clin Invest 2012; 122: 327–336. 22156198 10.1172/JCI57990 PMC3248287

[91] Nadkarni S , Smith J , Sferruzzi‐Perri AN , *et al* Neutrophils induce proangiogenic T cells with a regulatory phenotype in pregnancy. Proc Natl Acad Sci U S A 2016; 113: E8415–E8424. 27956610 10.1073/pnas.1611944114 PMC5206541

[92] Eruslanov EB . Phenotype and function of tumor‐associated neutrophils and their subsets in early‐stage human lung cancer. Cancer Immunol Immunother 2017; 66: 997–1006. 28283697 10.1007/s00262-017-1976-0 PMC5522629

[93] Hellebrekers P , Vrisekoop N , Koenderman L . Neutrophil phenotypes in health and disease. Eur J Clin Invest 2018; 48(suppl 2): e12943. 29682724 10.1111/eci.12943 PMC6282827

[94] Gabrilovich DI . Myeloid‐derived suppressor cells. Cancer Immunol Res 2017; 5: 3–8. 28052991 10.1158/2326-6066.CIR-16-0297 PMC5426480

[95] Zilio S , Serafini P . Neutrophils and granulocytic MDSC: the Janus God of Cancer immunotherapy. Vaccines (Basel) 2016; 4: E31. 27618112 10.3390/vaccines4030031 PMC5041025

[96] Fridlender ZG , Sun J , Kim S , *et al* Polarization of tumor‐associated neutrophil phenotype by TGF‐beta: “N1” versus “N2” TAN. Cancer Cell 2009; 16: 183–194. 19732719 10.1016/j.ccr.2009.06.017 PMC2754404

[97] Kumar V , Patel S , Tcyganov E , *et al* The nature of myeloid‐derived suppressor cells in the tumor microenvironment. Trends Immunol 2016; 37: 208–220. 26858199 10.1016/j.it.2016.01.004 PMC4775398

[98] Leliefeld PH , Koenderman L , Pillay J . How neutrophils shape adaptive immune responses. Front Immunol 2015; 6: 471. 26441976 10.3389/fimmu.2015.00471 PMC4568410

[99] Eruslanov EB , Singhal S , Albelda SM . Mouse versus human neutrophils in cancer: a major knowledge gap. Trends Cancer 2017; 3: 149–160. 28718445 10.1016/j.trecan.2016.12.006 PMC5518602

[100] Zhou SL , Zhou ZJ , Hu ZQ , *et al* Tumor‐associated neutrophils recruit macrophages and T‐regulatory cells to promote progression of hepatocellular carcinoma and resistance to sorafenib. Gastroenterology 2016; 150: 1646–1658 e1617. 26924089 10.1053/j.gastro.2016.02.040

[101] Eruslanov E , Neuberger M , Daurkin I , *et al* Circulating and tumor‐infiltrating myeloid cell subsets in patients with bladder cancer. Int J Cancer 2012; 130: 1109–1119. 21480223 10.1002/ijc.26123

[102] Mishalian I , Bayuh R , Eruslanov E , *et al* Neutrophils recruit regulatory T‐cells into tumors via secretion of CCL17 – a new mechanism of impaired antitumor immunity. Int J Cancer 2014; 135: 1178–1186. 24501019 10.1002/ijc.28770

[103] Governa V , Trella E , Mele V , *et al* The interplay between neutrophils and CD8(+) T cells improves survival in human colorectal cancer. Clin Cancer Res 2017; 23: 3847–3858. 28108544 10.1158/1078-0432.CCR-16-2047

[104] Singhal S , Bhojnagarwala PS , O'Brien S , *et al* Origin and role of a subset of tumor‐associated neutrophils with antigen‐presenting cell features in early‐stage human lung cancer. Cancer Cell 2016; 30: 120–135. 27374224 10.1016/j.ccell.2016.06.001 PMC4945447

[105] Eruslanov EB , Bhojnagarwala PS , Quatromoni JG , *et al* Tumor‐associated neutrophils stimulate T cell responses in early‐stage human lung cancer. J Clin Invest 2014; 124: 5466–5480. 25384214 10.1172/JCI77053 PMC4348966

[106] Hiramatsu S , Tanaka H , Nishimura J , *et al* Neutrophils in primary gastric tumors are correlated with neutrophil infiltration in tumor‐draining lymph nodes and the systemic inflammatory response. BMC Immunol 2018; 19: 13. 29661142 10.1186/s12865-018-0251-2 PMC5902874

[107] Brackett CM , Gollnick SO . Photodynamic therapy enhancement of anti‐tumor immunity. Photochem Photobiol Sci 2011; 10: 649–652. 21253659 10.1039/c0pp00354a PMC3197776

[108] Kousis PC , Henderson BW , Maier PG , *et al* Photodynamic therapy enhancement of antitumor immunity is regulated by neutrophils. Cancer Res 2007; 67: 10501–10510. 17974994 10.1158/0008-5472.CAN-07-1778 PMC2919236

[109] Tomay F , Wells K , Duong L , *et al* Aged neutrophils accumulate in lymphoid tissues from healthy elderly mice and infiltrate T‐ and B‐cell zones. Immunol Cell Biol 2018; 96: 831–840. 29603362 10.1111/imcb.12046

[110] Cross A , Bakstad D , Allen JC , *et al* Neutrophil gene expression in rheumatoid arthritis. Pathophysiology 2005; 12: 191–202. 16112850 10.1016/j.pathophys.2005.07.006

[111] Cross A , Bucknall RC , Cassatella MA , *et al* Synovial fluid neutrophils transcribe and express class II major histocompatibility complex molecules in rheumatoid arthritis. Arthritis Rheum 2003; 48: 2796–2806. 14558085 10.1002/art.11253

[112] Bird AK , Chang M , Barnard J , *et al* Neutrophils slow disease progression in murine lupus via modulation of autoreactive germinal centers. J Immunol 2017; 199: 458–466. 28584005 10.4049/jimmunol.1700354 PMC5524201

[113] Corsiero E , Bombardieri M , Carlotti E , *et al* Single cell cloning and recombinant monoclonal antibodies generation from RA synovial B cells reveal frequent targeting of citrullinated histones of NETs. Ann Rheum Dis 2016; 75: 1866–1875. 26659717 10.1136/annrheumdis-2015-208356 PMC5036240

[114] Corsiero E , Pratesi F , Prediletto E , *et al* NETosis as source of autoantigens in rheumatoid arthritis. Front Immunol 2016; 7: 485. 27895639 10.3389/fimmu.2016.00485 PMC5108063

[115] Papayannopoulos V . Neutrophil extracellular traps in immunity and disease. Nat Rev Immunol 2018; 18: 134–147. 28990587 10.1038/nri.2017.105

[116] Gestermann N , Di Domizio J , Lande R , *et al* Netting neutrophils activate autoreactive B cells in lupus. J Immunol 2018; 200: 3364–3371. 29632142 10.4049/jimmunol.1700778

[117] Lande R , Ganguly D , Facchinetti V , *et al* Neutrophils activate plasmacytoid dendritic cells by releasing self‐DNA‐peptide complexes in systemic lupus erythematosus. Sci Transl Med 2011; 3: 73ra19. 10.1126/scitranslmed.3001180 PMC3399524 21389263

[118] Garcia‐Romo GS , Caielli S , Vega B , *et al* Netting neutrophils are major inducers of type I IFN production in pediatric systemic lupus erythematosus. Sci Transl Med 2011; 3: 73ra20. 10.1126/scitranslmed.3001201 PMC3143837 21389264

[119] Kessenbrock K , Krumbholz M , Schonermarck U , *et al* Netting neutrophils in autoimmune small‐vessel vasculitis. Nat Med 2009; 15: 623–625. 19448636 10.1038/nm.1959 PMC2760083

[120] Diana J , Simoni Y , Furio L , *et al* Crosstalk between neutrophils, B‐1a cells and plasmacytoid dendritic cells initiates autoimmune diabetes. Nat Med 2013; 19: 65–73. 23242473 10.1038/nm.3042

[121] Zhang H , Wang S , Huang Y , *et al* Myeloid‐derived suppressor cells are proinflammatory and regulate collagen‐induced arthritis through manipulating Th17 cell differentiation. Clin Immunol 2015; 157: 175–186. 25680967 10.1016/j.clim.2015.02.001 PMC4657752

[122] Wu H , Zhen Y , Ma Z , *et al* Arginase‐1‐dependent promotion of TH17 differentiation and disease progression by MDSCs in systemic lupus erythematosus. Sci Transl Med 2016; 8: 331ra340. 10.1126/scitranslmed.aae0482 PMC4895207 27009269

[123] Zhu J , Chen S , Wu L , *et al* The expansion of myeloid‐derived suppressor cells is associated with joint inflammation in rheumatic patients with arthritis. Biomed Res Int 2018; 2018: 5474828. 30046600 10.1155/2018/5474828 PMC6038685

[124] Vlachou K , Mintzas K , Glymenaki M , *et al* Elimination of granulocytic myeloid‐derived suppressor cells in lupus‐prone mice linked to reactive oxygen species‐dependent extracellular trap formation. Arthritis Rheumatol 2016; 68: 449–461. 26414650 10.1002/art.39441

[125] Fujii W , Ashihara E , Hirai H , *et al* Myeloid‐derived suppressor cells play crucial roles in the regulation of mouse collagen‐induced arthritis. J Immunol 2013; 191: 1073–1081. 23804709 10.4049/jimmunol.1203535

[126] Ioannou M , Alissafi T , Lazaridis I , *et al* Crucial role of granulocytic myeloid‐derived suppressor cells in the regulation of central nervous system autoimmune disease. J Immunol 2012; 188: 1136–1146. 22210912 10.4049/jimmunol.1101816

[127] Guan Q , Moreno S , Qing G , *et al* The role and potential therapeutic application of myeloid‐derived suppressor cells in TNBS‐induced colitis. J Leukoc Biol 2013; 94: 803–811. 23901119 10.1189/jlb.0113050

[128] Haile LA , von Wasielewski R , Gamrekelashvili J , *et al* Myeloid‐derived suppressor cells in inflammatory bowel disease: a new immunoregulatory pathway. Gastroenterology 2008; 135: 871–881 881.e871–875. 18674538 10.1053/j.gastro.2008.06.032

[129] Crook KR , Liu P . Role of myeloid‐derived suppressor cells in autoimmune disease. World J Immunol 2014; 4: 26–33. 25621222 10.5411/wji.v4.i1.26 PMC4302755

[130] Yi H , Guo C , Yu X , *et al* Mouse CD11b+Gr‐1+ myeloid cells can promote Th17 cell differentiation and experimental autoimmune encephalomyelitis. J Immunol 2012; 189: 4295–4304. 23034169 10.4049/jimmunol.1200086 PMC3478426

[131] Guo C , Hu F , Yi H , *et al* Myeloid‐derived suppressor cells have a proinflammatory role in the pathogenesis of autoimmune arthritis. Ann Rheum Dis 2016; 75: 278–285. 25371442 10.1136/annrheumdis-2014-205508 PMC4418961

